# Aroclor1254 disrupts the blood–testis barrier by promoting endocytosis and degradation of junction proteins via p38 MAPK pathway

**DOI:** 10.1038/cddis.2017.224

**Published:** 2017-05-25

**Authors:** Xiaoyu Jia, Ying Xu, Weixing Wu, Yunxia Fan, Guoli Wang, Tianbiao Zhang, Wenhui Su

**Affiliations:** 1 Department of Biochemistry and Molecular Biology, College of Basic Medical Science, China Medical University, Shenyang 110001, China

## Abstract

The blood–testis barrier (BTB) constituted by coexisting junction apparatus between Sertoli cells (SCs) plays an important role in spermatogenesis, which is a known target of various environmental toxicants. The commercial polychlorinated biphenyls mixture, Aroclor1254, has been shown to impair male reproduction by decreasing sperm count and affecting SC metabolism. This study was designed to investigate the effects of Aroclor1254 on the BTB integrity and elucidate the underlying mechanisms. We found that Aroclor1254 treatment in rats (1 or 3 mg/kg per day for 21 consecutive days) and in primary cultured SCs (5 or 10 *μ*g/ml for 48 h) could induce BTB disruption via p38 MAPK pathway, concurrently with increments in junction proteins (JAM-A, N-cadherin, and *β*-catenin) endocytosis, and occludin ubiquitination. Either inhibition of caveolin-dependent membrane protein internalization by cholesterol oxidase or silencing E3 ubiquitine ligase Itch by small interfering RNA could partially counteract the effects of Aroclor1254 on the barrier function of cultured SCs. These results demonstrate that Aroclor1254 disrupts the BTB function by promoting the caveolin-dependent endocytosis and ubiquitine–proteasome degradation of junction proteins through the p38 MAPK pathway, which might be the potential reasons for its negative effects on spermatogenesis and male reproduction.

Polychlorinated biphenyls (PCBs) are a group of environmental endocrine-disrupting compounds, which are widely dispersed as a result of industrial and manufacturing activities.^1^ Although the industrial production of PCBs has been prohibited since the late 1970s, their accumulation in nature and food chains still pose health threat to humans due to their lipophilicity, chemical stability, and especially their tendency to accumulate in steroid hormone-producing organs.^2, 3^ It has been revealed in several species that PCBs had detrimental effects on male reproductive functions and could result in reduced testis weight and sperm count.^4, 5^ The epidemiologic studies in human also found the negative correlation between the levels of serum PCBs and testosterone.^6^ However, the specific effects and mechanisms of PCBs on different stages of spermatogenesis still remain poorly elucidated.

The blood–testis barrier (BTB), constituted by coexisting tight junction (TJ), basal ectoplasmic specialization (ES, a testis-specific adherens junction), and desmosome-like junctions between adjacent Sertoli cells (SCs), provides an individual environment for the development of spermatocytes. The turnover and interactions of the constituted proteins at these junctions contribute much to the BTB integrity and its physiological dynamics,^7, 8^ which might also undergo restructuring or disruption when exposed to exogenous detriments.^9, 10, 11, 12, 13^ Existing studies have proved that PCBs could impair testicular physiology by generating reactive oxygen species in rat SCs.^14, 15^ The prenatal exposure to 3,3′,4,4′,5-pentachlorobiphenyl (PCB126) was also reported to induce defective spermatogenesis and renewed SC proliferation in adult rats.^16, 17^ Nevertheless, little work has been done to investigate the possible influence of PCBs on the BTB integrity, which might account much for their impairments on spermatogenesis. In this study, we explored the effects of PCBs on BTB integrity by intraperitoneal injection of Aroclor1254, the frequently used commercial PCBs mixture, which contains 54% chlorine (mass percent), in rats or treating primary cultured SCs with sub-cytotoxic doses of Aroclor1254.

The mitogen-activated protein kinase (MAPK) signaling functions as a key role in various cellular events, which was also involved in numerous male reproductive processes including spermatogenesis, spermiation, capacitation, and acrosome reaction before fertilization.^18^ Recent literatures have reported the involvement of p38 MAPK and ERK1/2 signaling pathway in the adverse effects of some environmental toxicants including the PCBs.^19, 20, 21^ Herein, we detected the variation of p38 activity during Aroclor1254 contamination and further investigated its role in BTB function damage induced by Aroclor1254.

Therefore, the aim of this study was to identify the influence of Aroclor1254 on BTB function and the underlying intracellular signaling pathways. Our results revealed the enhanced endocytosis of several junction proteins associated with an increased caveolin-1 level and the enhanced ubiquitination of TJ protein occludin by the E3 ligase Itch after Aroclor1254 administration in primary cultured SCs, both of which were dependent on an elevated p38 activity and might be the potential mechanisms for Aroclor1254-induced BTB disruption and spermatogenesis disturbance.

## Results

### Aroclor1254 disrupted BTB integrity via p38 MAPK signaling pathway *in vivo*

**Figure 1:**
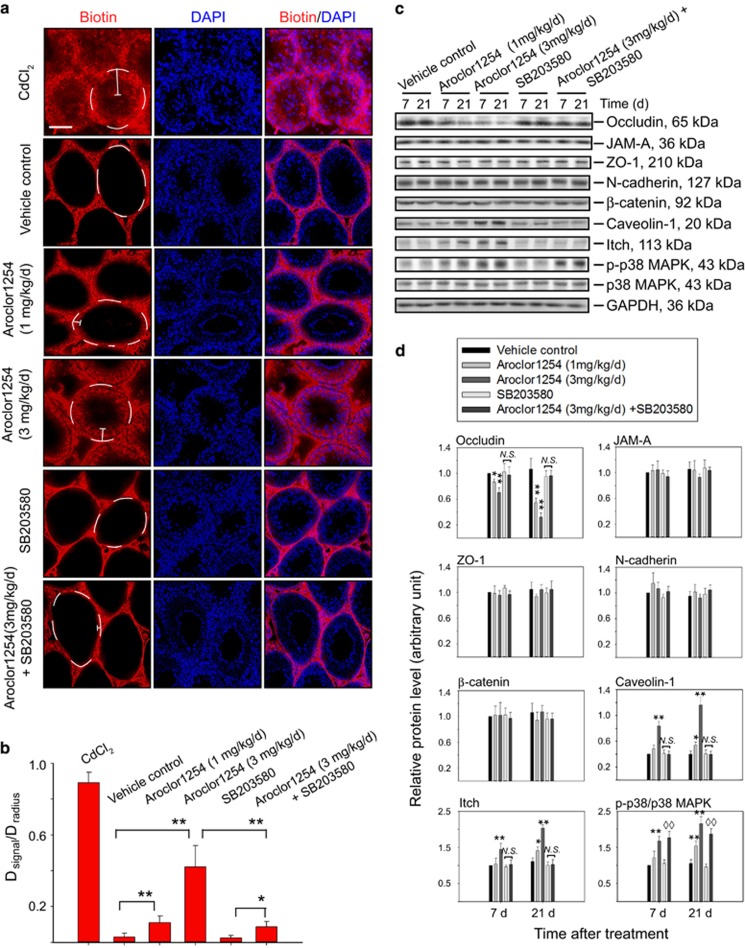
Disruption of the BTB by Aroclor1254 via p38 MAPK in the rat testis. Male rats were administered intraperitoneally with corn oil control, Aroclor1254, SB203580, or the mixture of Aroclor1254 and SB203580 as described in ‘Materials and methods’ section. (**a**) BTB integrity assay *in vivo*. EZ-Link Sulfo-NHS-LC-Biotin was injected into the testis interstitium, the distribution of which was observed using specific Alexa Fluor 568-conjugated streptavidin (red). Cell nuclei were stained with DAPI (blue). For positive control, rats were treated with a single dose of CdCl_2_ at 5 mg/kg 3 days before BTB integrity assay. Disruption of the BTB was reflected by diffusion distance (denoted by the white segments) of the indicator from the basal lamina (denoted by the white broken circles) into the tubule lumen. Scale bar=80 *μ*m, which applied to all micrographs in (**a**). (**b**) Histogram illustrating results of the BTB integrity assay. The permeability of BTB was semi quantified by the ratio of diffusion distance of the indicator (*D*_signal_) and the radius of the corresponding tubule (*D*_radius_). Each bar refers to mean±S.D. of 80 tubules randomly selected from four rats. **P<0.01. (**c**) Immunoblot analysis of BTB proteins with GAPDH serving as a protein loading control. The treated animals were killed after 7 or 21 days and testis lysates were prepared using tissue incised within 4 mm from the entry point of Aroclor1254. (**d**) Histograms summarizing the result shown in **c** after each data point was normalized against GAPDH except that the level of p-p38 MAPK was normalized to total p38 MAPK expression. Protein level of the vehicle control group on day 7 was arbitrarily set at 1. Each bar refers to mean±S.D. of *n*=3 rats. *P<0.05; **P<0.01, both compared with the vehicle control group. ^◊◊^*P*<0.01, compared with the SB203580 group. NS, no significant difference; p-p38, phosphorylated p38

The impact of Aroclor1254 on BTB integrity in rats was assessed using Sulfo-NHS-LC-Biotin as the indicator. After intraperitoneal injection of Aroclor1254 for 21 consecutive days, the biotin signal (red) was found to permeate into the seminiferous epithelium to different distance depending on the administration dosage as compared with the distinct ring of biotin that encircled the tubules in vehicle group, indicating a compromise of BTB integrity (Figures 1a and b). However, in the group administered simultaneously with Aroclor1254 and SB203580, the known p38 MAPK inhibitor, the penetration of the indicator was much weakened, which illustrated the participation of p38 MAPK pathway during the disruption of BTB by Aroclor1254 (Figures 1a and b). This was further validated by the following immunoblot analysis, which displayed an obvious elevation of the phosphorylated p38 in Aroclor1254 groups (Figures 1c and d).

To investigate the effects of Aroclor1254 on BTB structure, we detected the levels of several junction proteins and a significant decrease of occludin in the testis lysate was revealed (Figures 1c and d), which was also confirmed by immunofluorescence (Supplementary Figure 1). Although no obvious change was found in the levels of other junction proteins, immunostaining exhibited broader and more indistinct fluorescent signals when JAM-A and N-cadherin were detected after Aroclor1254 exposure (Supplementary Figure 1). What interested us then was the upregulation of Itch, the E3 ubiquitine ligase, which was known to regulate the turnover of occluding (Figures 1c and d).^22, 23^ Moreover, the level of caveolin-1, the key structural marker of caveolae was also induced in rats exposed to Aroclor1254 (Figures 1c and d). Furthermore, consistent with the BTB assay, additional p38 inhibitor could counteract the impacts of Aroclor1254 on the steady-state levels and localizations of the above proteins (Figures 1c and d; Supplementary Figure 1). Therefore, it could be hypothesized that Aroclor1254 might influence the BTB function by accelerating endocytosis and proteasome degradation of junction proteins via the p38 MAPK pathway.

### Aroclor1254 decreased barrier function of the SC epithelium *in vitro* via p38 MAPK

**Figure 2:**
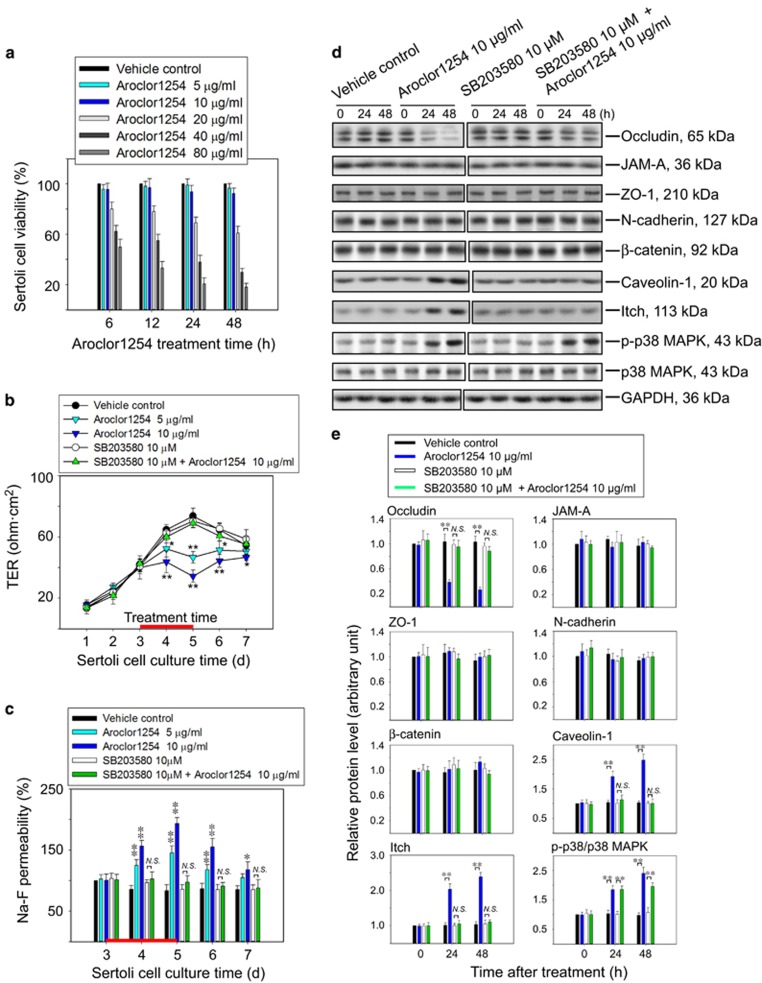
Decreased barrier function of the SC epithelium after Aroclor1254 treatment via p38 MAPK. Sertoli cells were isolated from 20-day-old rat testis and cultured for 7 days. (**a**) Cell viability assay to detect cytotoxicity of Aroclor1254 on primary Sertoli cells. SCs cultured at 0.5 × 10^6^/cm^2^ were treated with different concentrations of Aroclor1254 (0–80 *μ*g/ml) for 6, 12, 24, and 48 h from day 3 before an enhanced CCK-8 kit was used and absorbance at 450 nm was recorded for cell viability calculating. Aroclor1254 over 20 *μ*g/ml could induce significant decrease in SCs viability. (**b** and **c**) Effects of Aroclor1254 on the barrier function of SC by recording TER (**b**) or measuring the permeability of Na-F (**c**) across the cell epithelium. The Na-F permeability in the vehicle control group on day 3 was arbitrarily set at 100%. Aroclor1254 below cytotoxic concentrations was found to perturb the SC barrier function. *P<0.05; **P<0.01, compared with the vehicle control group at the corresponding time point. (**d**) Immunoblot analysis to assess the levels of different BTB proteins in SC lysates prepared at different time points after Aroclor1254 treatment with GAPDH serving as a loading control. (**e**) Histograms summarizing the result shown in **d** after each data point was normalized against GAPDH except that the level of p-p38 MAPK was normalized to total p38 MAPK expression. Protein level of the vehicle control group at time 0 was arbitrarily set at 1. Each bar refers to mean±S.D. of *n*=3 experiments using different batches of SC lysates. **P<0.01. NS, no significant difference; p-p38, phosphorylated p38

To confirm whether the effect of Aroclor1254 on the BTB *in vivo* was due to its influence on SCs, we treated primary cultured SCs with Aroclor1254 *in vitro*. Two concentrations of Aroclor1254 (i.e., 5 or 10 *μ*g/ml) were selected according to the result of cell viability assay with negligible cytotoxicity, which might affect the detection of epithelial barrier functions (Figure 2a). Daily measurement of the transepithelial electrical resistance (TER) value revealed declining barrier functions induced by Aroclor1254, by more than 30% in the low-dose group and 50% in the high-dose group, respectively, after 48-h treatment (Figure 2b). The permeability of the SC epithelium to Na-F was also induced significantly in Aroclor1254 groups (Figure 2c). After withdrawing Aroclor1254, a restoration of barrier functions was observed on culture day 6 and 7 (Figures 2b and c). Moreover, the counteraction of p38 inhibitor against Aroclor1254 on barrier function was more apparent in this *in vitro* experiment than the above *in vivo* BTB assay, which showed a completely recovering of both the TER value and the Na-F permeability in the Aroclor1254+SB203580 group (Figures 2b and c). Due to the known inhibition of SB203580 on protein kinase B (PKB), we also detected the phosphorylation level of PKB on Ser473 and Thr308 after Aroclor1254 treatment, which revealed no significant changes (Supplementary Figures 2a and b).

**Figure 3:**
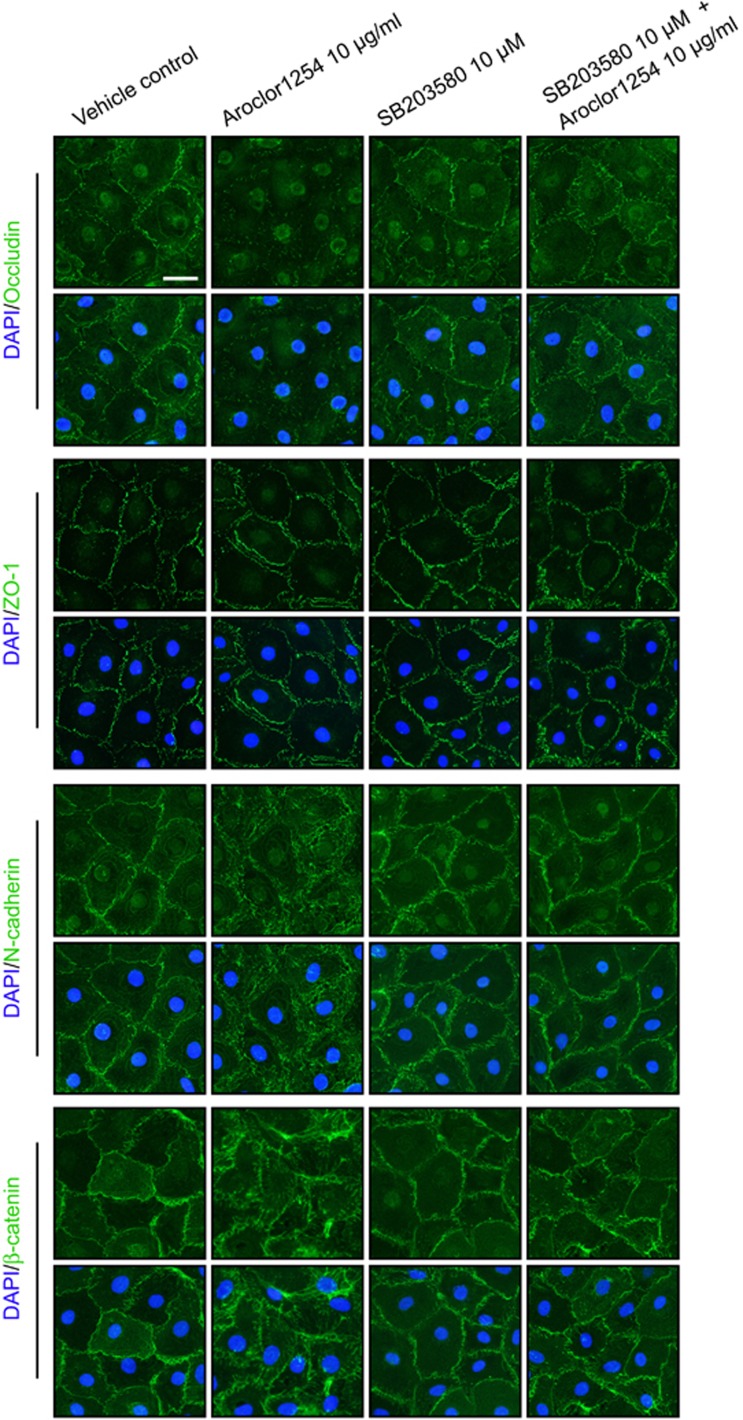
Aroclor1254 influenced distribution of junction proteins in primary SCs. Primarily isolated SCs were cultured on matrigel-coated coverslips at 0.05 × 10^6^/cm^2^. On day 3, Aroclor1254 (10 *μ*g/ml), SB203580 (10 *μ*m), or the mixture of the above two was added to the medium. After 24 h, the cells were fixed with 4% paraformaldehyde. The distribution of BTB proteins at cell–cell junctions was observed by immunofluorescence using Alexa Fluor 488-conjugated secondary antibody (green) with cell nuclei stained with DAPI (blue). A reduced signal of occludin and disturbed distributions of N-cadherin and *β*-catenin were detected at cell junctions of Aroclor1254-treated SCs, as compared with the sharply defined signals in the control and the Aroclor1254+SB203580 groups. Scale bar=25 *μ*m, which applied to all micrographs in Figure 3

Consistent with the *in vivo* findings, the steady-state level of occludin in cultured SCs was downregulated in response to Aroclor1254 (Figures 2c and d), which was also reflected by immunofluorescence (Figure 3). As for other junction proteins detected, namely zonula occluden (ZO-1), N-cadherin, and *β*-catenin, no variations in protein level were perceived. Whereas the immunofluorescence displayed an abnormal and disturbed distribution of N-cadherin and *β*-catenin at cell junctions after Aroclor1254 treatment (Figure 3). Besides, the protein level of caveoline-1 and Itch in the SC lysates were both induced by Aroclor1254 (Figures 2c and d). The additional p38 inhibitor could rehabilitate the changed levels and distributions of the above proteins, which might explain the recovering of barrier function in the Aroclor1254+SB203580 group (Figures 2c and d; Figure 3).

### Aroclor1254–p38 MAPK pathway enhanced endocytosis of BTB junction proteins *in vitro*

**Figure 4:**
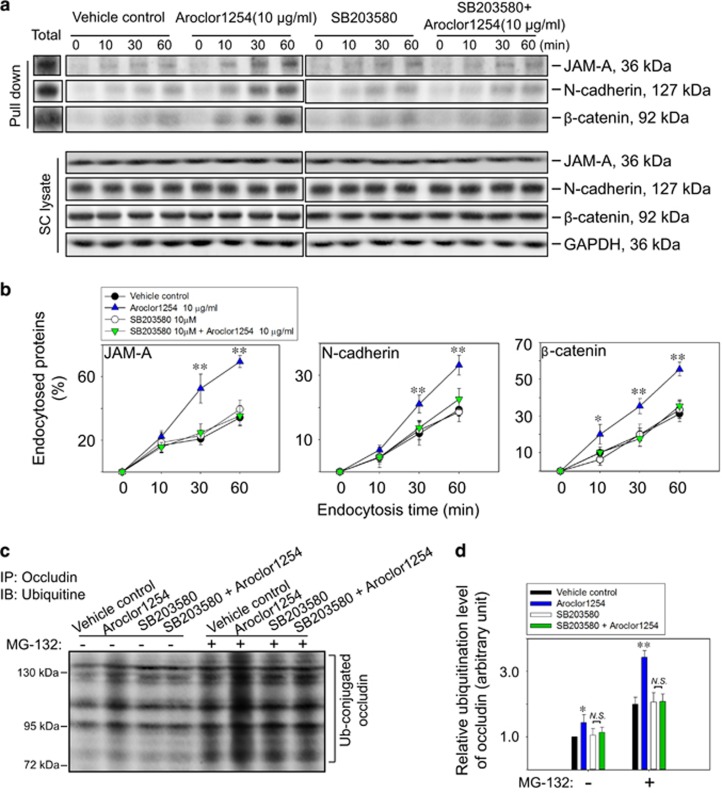
Aroclor1254 accelerated junction protein endocytosis and ubiquitination via p38 MAPK. (**a**) Immunoblot analysis of endocytosed JAM-A, N-cadherin, and *β*-catenin in SCs at different time points (0, 10, 30, 60 min) after cell surface biotinylation for 30 min in the presence of Aroclor1254 (or the mixture of Aroclor1254 and SB203580). The endocytosed proteins were pulled-down by UltraLink Immobilized NeutrAvidin Plus Resin after stripping residual biotin on cell membrane as described in ‘Materials and methods’ section. Total biotinylated proteins at 0 min without stripping were also detected as a positive control. Cell lysates without pull-down were also analyzed by immunoblot to confirm identical levels of the tested protein between groups with GAPDH served as the loading control. (**b**) Line and scatter graphs summarizing the result shown in **a** by calculating the percentage of endocytosed protein at each data point *versus* the total biotinylated protein. Each bar refers to mean±S.D. of *n*=3 independent experiments using SCs cultured from different rats. *P<0.05; **P<0.01, compared with the vehicle control at the same time point. (**c**) Co-immunoprecipitation assay using SCs lysates to detect the ubiquitination level of occludin after Aroclor1254 treatment or combined treatment of Aroclor1254 and SB20358 for 48 h. MG-132 of 50 *μ*m was applied to inhibit the proteasome degradation of ubiquitine-conjugated proteins. (**d**) Histogram summarizing the result shown in **c** with the ubiquitinized occludin in the vehicle control SCs without MG-132 treatment was arbitrarily set at 1. *P<0.05; **P<0.01, compared with the corresponding control group. NS, no significant difference between two indicated groups

Due to the importance of junction protein internalization during BTB restructuring or disruption,^24^ we proceeded to examine the endocytosis of junction proteins. It was shown in Figure 4a that the BTB integral proteins were internalized in a time-dependent manner in the control group, in contrast with a roughly double growth (at 30 min point for JAM-A and N-cadherin, and 10 min point for *β*-catenin) in the presence of 10 *μ*g/ml Aroclor1254 without influencing the steady-state levels of these proteins. The percentage of endocytosed occludin was also inspected, while no variation was found after Aroclor1254 treatment when each data were normalized to the whole-occludin level in each group (Supplementary Figures 3a and b). Moreover, SB203580 could greatly redeem the enhanced internalization caused by Aroclor1254 (Figures 4a and b). These data demonstrated the induction of junction proteins endocytosis during the BTB disruption induced by Aroclor1254 via p38 MAPK pathway.

### Aroclor1254-p38 MAPK pathway accelerated the ubiquitination of occludin in cultured SCs

In order to find out if the ubiquitine–proteosome sytem (UPS) was responsible for the Aroclor1254-induced occludin decreasing, we performed co-immunoprecipitation to examine the ubiquitination level of occludin in the SC lysate. It was noted that there was a slight increase in the ubiquitinated occludin level after Aroclor1254 administration (Figures 4c and d). In particular, a more obvious elevation of occludin ubiquitination was detected in the Aroclor1254-treated SCs after the addition of the proteasome inhibitor MG-132 (Figures 4c and d). To exclude the possibility of transcriptional regulation, we detected the mRNA level of occludin, which was revealed to be unchanged after Aroclor1254 treatment (Supplementary Figure 3c). The ubiquitination assay results shown by Figure 4 also illustrated the inhibition of SB203580 on the effects of Aroclor1254, which indicated that Aroclor1254-p38 MAPK pathway might affect the BTB function via the UPS-dependent degradation of occludin.

### Caveolin-dependent endocytosis was involved in the effects of Aroclor1254 on the BTB function

**Figure 5:**
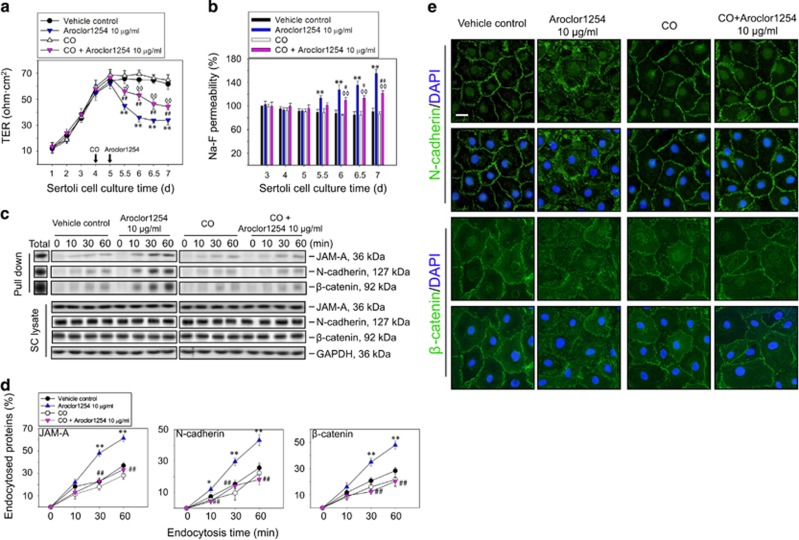
The effects of Aroclor1254 on the barrier function of SC epithelium after inhibiting the caveolin-dependent endocytosis. The SCs were treated with or without Aroclor1254 in the presence or absence of CO as the regimen depicted in Supplementary Figure S2a. (**a** and **b**) The barrier function of SC epithelium measured by recording TER (**a**) or calculating the permeability of Na-F (**b**) across the cell monolayer. The Na-F permeability in the vehicle control on day 3 was arbitrarily set at 100%. CO treatment was found to partially resist the disruption of BTB by Aroclor1254. Each bar refers to mean±S.D. of *n*=3 experiments using different batches of SCs. **P<0.01, compared with the vehicle control group. ^◊^*P*<0.05; ^◊◊^*P*<0.01, compared with the CO group. ^#^*P*<0.05; ^##^*P*<0.01, compared with the Aroclor1254 group. (**c**) Immunoblot analysis of endocytosed JAM-A, N-cadherin, and *β*-catenin in SCs at different time points (0, 10, 30, 60 min) after cell surface biotinylation for 30 min in the presence of Aroclor1254 (or the mixture of Aroclor1254 and CO). Total biotinylated proteins at 0 min without stripping served as the positive control. Cell lysates without pull-down were analyzed by immunoblot to confirm identical levels of the tested protein between groups with GAPDH as the loading control. (**d**) Line and scatter graphs summarizing the result shown in **c** by calculating the percentage of endocytosed protein at each data point *versus* the total biotinylated protein. The endocytosis of JAM-A, N-cadherin, and *β*-catenin showed no difference between the CO-treated cells and CO+Aroclor1254-treated cells. *P<0.05; **P<0.01, compared with the vehicle control group. ^##^*P*<0.01, compared with the Aroclor1254 group. (**e**) Immunofluorescence assay to observe the distribution of N-cadherin and *β*-catenin at SC–SC junctions on day 6 after Aroclor1254 treatment for 24 h in the presence or absence of CO, using Alexa Fluor 488-conjugated secondary antibody (green) with cell nuclei stained with DAPI (blue). Scale bar=25 *μ*m, which applied to all micrographs in (**e**)

To further confirm whether the increased level of caveolin-1 in SCs after Aroclor1254 treatment was responsible for the enhanced junction proteins endocytosis and decreased SC barrier function, we used cholesterol oxidase (CO) to inhibit the caveolin pathway in SC^25, 26^ (Supplementary Figure 4a). Immunoblot revealed that CO could not antagonize the Arolor1254-induced protein level changes, that is, the decreased occludin, increased caveolin-1, Itch, and phosphor-p38 MAPK levels (Supplementary Figures 4b and c). However, the disrupted barrier function reflected by the decreased TER value and increased Na-F permeability induced by Aroclor1254, though not fully restored, was partially rescued in the CO+Aroclor1254 group (Figures 5a and b). In addition, endocytosis assay confirmed that the increased endocytosis of JAM-A, N-cadherin, and *β*-catenin induced by Aroclor1254 were caveolae dependent, which could not be detected after the caveolin pathway was inhibited by CO (Figures 5c and d). In immunofluorescence assay, the disturbed localizations of N-cadherin and *β*-catenin at cell junctions could not be observed after Aroclor1254 treatment in the presence of CO (Figure 5e). These data illustrated the effects of Aroclor1254 on the BTB function were at least partially via caveolae-dependent endocytosis.

### Knockdown of E3 ligase Itch partially antagonized the BTB disruption caused by Aroclor1254

**Figure 6:**
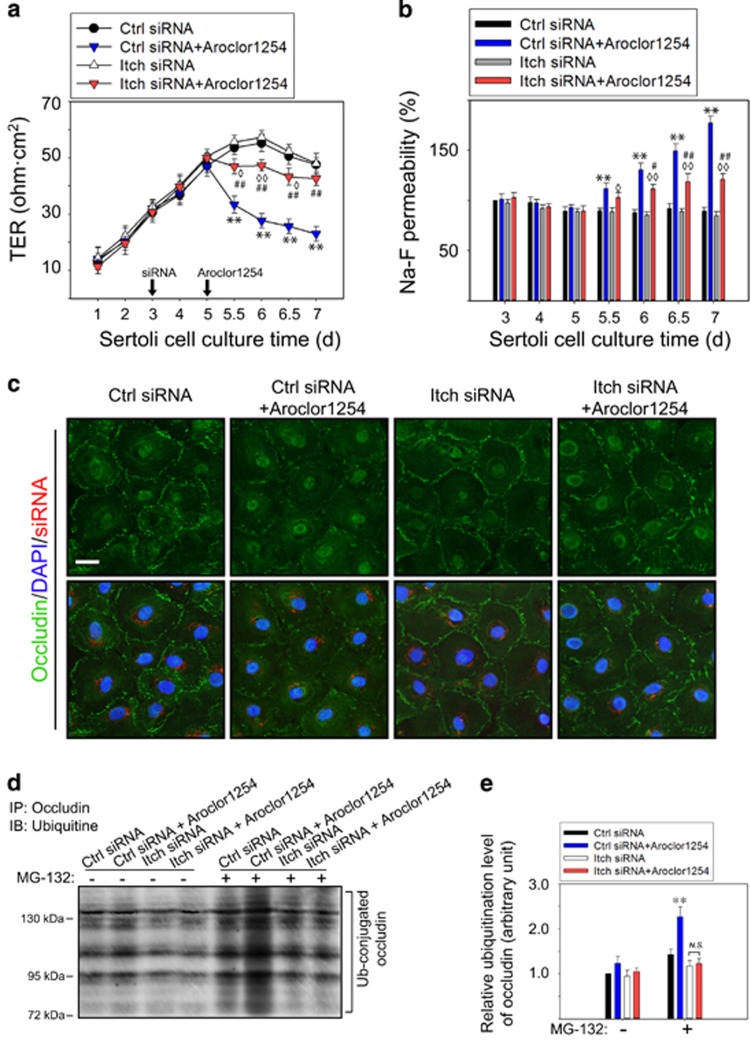
The effects of Aroclor1254 on the barrier function of SC epithelium after Itch knockdown. The SCs were treated with or without Aroclor1254 in the control siRNA or Itch siRNA-transfected SCs as the regimen depicted in Supplementary Figure S3a. (**a**) Barrier function assay *in vitro* by measuring the TER value of different groups on each culture day. The extent of barrier function decline in Itch-silenced SCs induced by Aroclor1254 was not as great as in the control siRNA-transfected cells. (**b**) Na-F permeability assay with the value in the Ctrl siRNA group on day 3 was arbitrarily set at 100%. Itch silencing partially antagonized the disruption of BTB by Aroclor1254. Each bar refers to mean±S.D. of *n*=3 experiments using different batches of SCs. **P<0.01, compared with the Ctrl siRNA group. ^◊^*P*<0.05; ^◊◊^*P*<0.01, compared with the Itch siRNA group. ^#^*P*<0.05; ^##^*P*<0.01, compared with the Ctrl siRNA+Aroclor1254 group. (**c**) Immunofluorescence assay to detect occludin (green) on day 6 after Aroclor1254 treatment for 24 h in the Itch siRNA or the control siRNA-transfected SCs with cell nuclei stained with DAPI (blue). The transfected siRNAs were indicated by siGLO-Red (red). Scale bar=25 *μ*m, which applied to all micrographs in (**e**). (**d**) Co-immunoprecipitation assay to examine the ubiquitination level of occludin after Aroclor1254 treatment in the Itch siRNA or the control siRNA-transfected SCs on day 7. MG-132 was used to inhibit the proteasome degradation of ubiquitinized proteins. (**e**) Histogram summarizing the result shown in (**d**), in which the ubiquitinized occludin level in the control siRNA-transfected SCs without MG-132 treatment was arbitrarily set at 1. **P<0.01, compared with the corresponding Ctrl siRNA group. NS, no significant difference between two indicated groups

Due to the upregulated level of Itch and occludin ubiquitination after Aroclor1254 treatment, we sought to investigate whether it was Itch that mediates the ubiquitination and proteasome degradation of occludin, which leads to the disruption of the BTB in response to Aroclor1254. In this sense, we transfected SCs with small interfering RNA (siRNA) targeting at Itch, followed by treatment of Aroclor1254 (Supplementary Figure 5a). Immunoblot analysis showed that when Itch was knocked down by ~80%, Aroclor1254 exposure could not induce the decline of occludin anymore, but the increments in phosphor-p38 MAPK and caveolin-1 still existed (Supplementary Figures 5b and c). This indicated that the ubiquitination and degradation of occludin might be only one of the reasons for Aroclor1254-induced BTB disruption. Then, the following TER measuring and Na-F permeability assay found a better barrier function in Itch-silencing SCs *versus* control siRNA-transfected SCs when opposed to Aroclor1254 (Figures 6a and b), though there remained a noticeable barrier function decrease when Itch-silencing SCs were treated with Aroclor1254 (Figures 6a and b). The resistance of Itch silencing on Aroclor1254-induced occludin decrease was also confirmed by immunofluorescence, which discovered no occludin intensity difference between Itch siRNA group and Itch siRNA+Aroclor1254 group (Figure 6c). Moreover, the increased ubiquitination and proteasome degradation of occludin mediated by Itch following Aroclor1254 treatment was further verified by the ubiquitination assay. No significant difference in the ubiquitinated occludin level was found between groups without MG-132 treatment. When the proteasome degradation was inhibited by MG-132, a noticeable increase in occludin ubiquitination was revealed after Aroclor1254 treatment in the control siRNA-transfected SCs, but not in the Itch siRNA-trasfected SCs (Figures 6d and e). These results confirmed the Aroclor1254-induced occludin decrease was mediated by the E3 ubiquitine ligase Itch and proteasome degradation, which accounts partially for the BTB disruption caused by Aroclor1254.

## Discussion

Endocrine disruptors have various estrogenic effects on male reproductive axis resulting in infertility.^27^ In the past decades, a number of epidemiological studies have assessed the influence of PCBs on male reproduction, which suggest that PCBs are related with reduced semen quality, sperm DNA damage, and lower serum testosterone levels.^28, 29, 30, 31^ SCs, which envelope the developing germ cells during spermatogenesis, are primary determinant of sperm number and one of the target sites of the endocrine disruptors in male reproductive tract. Previous studies using primarily cultured SCs or adult male rats found reduced antioxidant enzymes and increased cellular lipid peroxidation after Aroclor1254 treatment. The metabolic and secretary functions of SCs were also affected by Aroclor1254 as reflected by diminished androgen-binding protein and elevated lactate secretions.^14, 32^ Further investigations reported an increased caspase-8 expression and induction of SCs apoptosis in rats exposed to PCBs on account of the oxidative stress and Fas ligand activation.^15, 33^

Due to the key role of SCs in spermatogenesis and BTB integrity establishment, and the adverse impacts of PCBs on SCs functions, in this study we probed the effects of Aroclor1254, the commercial PCB mixture, on the SC barrier function both *in vivo* and *in vitro*. The present study in rats revealed the increase of BTB permeability induced by Aroclor1254 in a dose-dependent manner. The *in vitro*study herein using Aroclor1254 at doses without detectable cell toxicity also confirmed the compromise of the SC barrier functions, which showed a recovering tendency after Aroclor1254 removal, indicating a reversible disruption of the BTB integrity within the applied exposure dosage. Previous studies have illustrated the participation of ERK1/2 and p38 MAPK in junction restructuring within the seminiferous epithelium by affecting the turnover of junction proteins. For instance, the stimulated production of cadherins and occludin during BTB enhancement induced by testosterone was found to be accompanied with an increased p-ERK1/2 level in SCs.^34^ The ERK signaling pathway was also involved in the regulation of SC anchoring junctions and TJs by follicle-stimulating hormone.^35^ Besides, during the BTB restructuring or breakdown induced by cytokines or cadmium, the activated p38 MAPK cascade was associated with disruption of the TJ barrier and loss-of-junction proteins.^36, 37^ In this research, we detected an increased ratio of p-p38 MAPK/p38 MAPK after Aroclor1254 exposure, indicating the activation of p38 signaling. More importantly, the p38 pathway inhibitor SB2023580 could partially or almost completely rescue the barrier function loss caused by Aroclor1254 *in vivo* or *in vitro*, respectively, which strongly suggest the participation of p38 pathway during this process. On the other hand, the discrepancy of SB203580 in rescuing the barrier function between *in vivo* and *in vitro* studies might be due to the different signaling pathway affected by Aroclor1254 in different cell types *in vivo*. For example, the downregulated serum testosterone by Aroclor1254 injection might also affect the BTB assembly independent of the p38 signaling. It was known that many environmental pollutants, including PCBs, exert their adverse effects via the aryl hydrocarbon receptor, which has been proposed to interfere with different MAPK cascades.^38^ Our result was consistent with a recent study identifying an induced p38 activity, required for paxillin phosphorylation and focal adhesion assembly, during the dysregulation of vascular endothelial integrity induced by a quinone type of PCB.^19^ Although SB203580 was also known to inhibit PKB activity^39^ and Aroclor1254 has been reported to increase the phosphorylation of PKB in hepatocellular carcinoma cells,^40^ the phosphorylation level of PKB in cultured SCs did not show significant difference after Aroclor1254 exposure, which further confirmed the counteraction of SB203580 against Aroclor1254 herein was due to its inhibition on p38 MAPK.

In this study, variations of junction protein turnover at the BTB after Aroclor1254 treatment were reflected by a decreased occudin level and growing endocytosis of JAM-A, N-cadherin, and *β*-catenin. As a manner of post-translational modification, ubiquitination of proteins in the testis occurs in many stages of spermatogenesis, including the regulation of junction complexes in the seminiferous epithelium.^41^ In MDCK cells, the turnover of occludin was proved by yeast two-hybrid screening to be associated with the E3 ubiquitin ligase Itch.^23^ In primary SCs, the increased occludin level during TJ assembling at cell interface was proved to coincide with a reduction in Itch.^22^ Further study in SCs revealed the reciprocal regulation of occludin by Itch and the ubiquitin-conjugating enzyme UBC4 during the dibutyryl-cAMP-induced barrier function breakdown.^22^ In the light of these, we detected the level of Itch to confirm whether Itch-induced ubiquitination and proteasome degradation of occludin was involved in the BTB damage caused by Aroclor1254. In accordance with our hypothesis, the raised expression of Itch and increased ubiquitin-conjugated occludin in Aroclor1254-treated SCs, together with the unchanged occludin mRNA level, suggested a post-translational regulation of occludin bioavailability via the UPS. Moreover, further Itch silencing could partially recover the disrupted epithelium integrity caused by Aroclor1254. These data strongly support the thesis that the ubiquitination of occludin by Itch was a crucial process facilitating the BTB injury by Aroclor1254. It should also be noted that silencing of Itch in SCs alone didn’t elevate the TJ barrier function in the absence of Aroclor1254, which might indicate Itch-induced occludin ubiquitination does not exert its role during physiological dynamics of the BTB. In our result, the upregulation of Itch in SCs exposed to Aroclor1254 that enhanced occludin ubiquitination was shown to rely on the elevated p38 MAPK activity. This finding provided a novel relationship between Itch and p38 signaling as compared with a recent research, which found an activation of p38 in skin lesions of Itch-deficient mice due to the retardation in TGF-*β*-activated kinase 1-binding protein 1 ubiquitination.^42^

Membrane protein endocytosis, depending on clathrin-coated pit, caveolae, or micropinocytosis, plays a pivotal role in cell junction establishment and disruption in epithelia.^43, 44^ The crucial regulators of the BTB dynamics during spermatogenesis, that is, testosterone and cytokines (TNF*α*, TGF-*β*2/3), were proved to accelerate the endocytosis of junction proteins, including occludin, JAM-A, and N-cadherin, via a clathrin-dependent way in SCs, which could be prevented by phenylarsine oxide (PAO, the known clathrin-dependent endocytosis inhibitor), but not the caveolin pathway inhibitor CO.^7, 8^ In vascular endothelial cells, the roles of caveolin-1 during the toxic effects exerted by PCBs have been investigated by a series of studies, in which the increased caveolin-1 level caused by PCB77 or PCB126 was associated with the endothelial nitric oxide synthase phosphorylation, induction of cytochrome P450 1A1, vascular cell adhesion molecule-1, or Nrf2 signaling.^45, 46, 47, 48^ However, information of the relationship between PCBs and caveolae-dependent endocytosis in the testis is lacking. In this study, we observed an increased internalization of JAM-A, N-cadherin, and *β*-catenin after Aroclor1254 treatment in SCs. Importantly, counteraction of this phenomenon by the addition of CO, coupled with the upregulated caveolin-1 in Aroclor1254 groups, suggested an activation of the caveolae-dependent junction protein endocytosis, which might account partially for the BTB destabilization induced by Aroclor1254. Therefore, our results indicated a disparate way dependent on caveolae, and not clarthrin, for junction protein internalization in SCs exposed to the environmental toxicant PCBs, as compared with the physiological factors such as testosterone and cytokines.

It is known that p38 MAPK acts as the core of stress-induced signaling as well as JNK and participates in endocytosis via phosphorylating components of the endocytic system.^49^ Nevertheless, in some studies in endothelial cells, p38 activation was shown to be the downstream event of the caveolae-dependent endocytosis. For instance, caveolin-1 was proved to interact with p38 and facilitate its phosphorylation in rat lung microvessel endothelial cells, which contributed to maintain endothelial monolayer integrity.^50^ In vascular endothelial cells, the PCB77-induced upregulation of monocyte chemoattractant protein-1 was demonstrated to be dependent on caveolin-1 and could also be prevented by inhibiting p38 activity.^51^ In our study, the signaling mechanism that associates Aroclor1254 to the increment of caveolin-1 was verified to be the elevated p38 activity and could be antagonized by SB203580. In addition, although the barrier function loss in SCs exposed to Aroclor1254 could be partially rescued by the inhibitor of caveolae-dependent endocytosis, it could not counteract the effect of Aroclor1254 on p38 phosphorylation (Supplementary Figure 4), suggesting p38 be an upstream regulator of caveolae during this process. Despite that the in-depth pathway of how p38 in regulation of the caveolin-1 level in SCs still remains to be fully elucidated, a recent study in nucleus pulposus cells revealed a blocking effect of the p38 MAPK pathway inhibitor on the enhanced caveolin-1 promotor activity during cytokines-induced apoptosis.^52^ These data suggest discrepant interactions between p38 MAPK and caveolae-dependent endocytosis, which might be a mutual influencing process like a positive feedback or completely irrelevant pathways under different outer stimuli and needs further investigations.

In conclusion, we provide evidence that the PCBs congener Aroclor1254 could disrupt the BTB integrity in rat via activating the p38 MAPK pathway. Two mechanisms regarding junction protein turnover were illustrated in this process, that is, the Itch-induced occludin ubiquitination and proteasome degradation, and the caveolae-dependent endocytosis of junction proteins (JAM-A, N-cadherin, and *β*-catenin), both of which led to the instability of junction apparatus between adjacent SCs and a subsequent damaged BTB. These findings might be the potential prelude for the known toxic effects of PCBs on male reproduction.

## Materials and methods

### Antibodies and reagents

The following commercial antibodies were applied according to the manufactures instructions for immunoblotting, immunoprecipitation, and immunofluorescence analysis in this study: rabbit anti-occludin (Invitrogen, Carsland, CA, USA; cat#71-1500), rabbit anti-junctional adhesion molecule (JAM)-A (Invitrogen, cat#36-1700), rabbit anti-ZO-1 (Invitrogen, cat#61-7300), rabbit anti-N-cadherin (Santa Cruz Biotechnology, Santa Cruz, CA, USA; cat#sc-7939), and rabbit anti-ubiquitine (Santa Cruz Biotechnology, cat#sc-9133), rabbit anti-*β* catenin (Invitrogen, cat#71-2700), rabbit anti-caveolin-1 (Invitrogen, cat#PA1-064), mouse anti-itch (BD Transduction Laboratories, Franklin Lakes, NJ, USA; cat#611198), rabbit anti-p38 (Cell Signaling Technology, Danvers, MA, USA;cat#9212), rabbit anti-phospho-p38 MAPK (Cell Signaling Technology, cat#9211), mouse anti-reduced glyceraldehyde-phosphate dehydrogenase (GAPDH) (Santa Cruz Biotechnology, cat#sc-32233), mouse anti-Akt1/2/3 (Santa Cruz Biotechnology, cat#sc-81434), mouse anti-phospho-Ser473-Akt1/2/3 (Santa Cruz Biotechnology, cat#sc-514032), mouse anti- phospho-Ser473-Akt1/2/3 (Santa Cruz Biotechnology, cat#sc-271966), HRP-conjugated bovine anti-rabbit IgG (Santa Cruz Biotechnology, cat#sc-2370), HRP-conjugated bovine anti-mouse IgG (Santa Cruz Biotechnology, cat#sc-2371), Alexa Fluor 488- or Alexa Fluor 555-conjugated donkey anti-rabbit IgG (Invitrogen, cat#A21206 or #A31572).

Aroclor1254 was purchased from Supleco (Bellefonte, PA, USA). SB203580 was supplied by Selleck Company (Houston, TX, USA). Protein A/G plus agarose, EZ-Link Sulfo-NHS-LC-Biotin, Sulfo-NHS-SS-Biotin, and UltraLink Immobilized NeutrAvidin Plus Resin were from Pierce Biotechnology (Rockford, IL, USA). Alexa Fluor 568-conjugated streptavidin, Prolong Gold Antifade reagent, Opti-MEM Reduced-Serum Medium, and Trizol reagent were from Life Invitrogen (Carsland, CA, USA). DMEM/F12 medium was from Gibco (Gaithersburg, MD, USA). Ribojuice siRNA transfection reagent was from EMD Biosciences (San Diego, CA, USA). SiRNAs were designed and synthesized by GenePharma (Shanghai, China). MG-132 was the product of Calbiochem (Darmstadt, Hessen, Germany). M-MLV reverse transcriptase was from Promega (Madison, WI, USA). QuantiTect SYBR Green PCR Kit was product of Qiagen (Dusseldorf, Germany). Other reagents, unless otherwise specified, were all products of Sigma-Aldrich (St. Louis, MO, USA).

### Animals and treatment

The use and treatment procedures of 20-day- and 90-day-old male Sprague–Dawley rats from the Department of Laboratory Animals of China Medical University (CMU) were approved by the CMU Animal Care and Use Committee. Animals were raised in a light:dark cycle of 12 h:12 h at 20–22 °C with free access to standard chow and water, and treated humanely to alleviate distress and discomfort. The protocol for animal handling and treatment was approved by the CMU Animal Care and Use Committee.

In the *in vivo* experiments, 90-day-old adult Sprague–Dawley rats weighting 250–280 g were injected with Aroclor1254 (diluted in corn oil) intraperitoneally at 1 or 3 mg/kg (body weight) per day for 21 consecutive days. The control groups were injected with isometric corn oil vehicle. Some animals of the control and the 3 mg/kg per day Aroclor1254 group were injected simultaneously with SB203580 at 10 mg/kg every 3 days. These doses and duration time were determined according to previous literatures and our pilot experiments.^4, 14^

### BTB integrity assay *in vivo*

Biotin was used as an indicator in the BTB integrity assay.^21, 53^ Briefly, three to four rats from each group mentioned above were anesthetized with ketamine HCl. Then the testes were exposed and small openings were made with ophthalmic forceps. Thereafter, 50 *μ*l EZ-Link Sulfo-NHS-LC-Biotin (10 mg/ml in PBS containing 1 mM CaCl_2_) was injected into the testis interstitium. After a duration of 30 min, the animals were killed by CO_2_ asphxiation and the testes were extracted and frozen in liquid nitrogen immediately. Then cryosections of 10 *μ*m were prepared at −20 °C, followed by fixation in 4% paraformaldehyde for 5 min. After being blocked with 5% bovine serum albumin diluted in PBS, the sections were incubated with Alexa Fluor 568-conjugated streptavidin at 1 : 200 for 1 h at room temperature. Then the sections were observed by fluorescence microscopy after mounting. The BTB integrity was evaluated by its ability to prevent the diffusion of biotin from the basal membrane toward the tubule lumen direction, which was semiquantitatively analyzed by plotting the distance of the fluorescence signal *versus* the corresponding radius of each tubule. Rats treated with a single dose of cadmium chloride (5 mg/kg) intraperitoneally 3 days before BTB integrity assay were used as positive controls, due to its known destructive effects on BTB.^9, 54^

### Primary cultures and treatment of SCs

SCs were isolated from 20-day-old rat testes according to a widely used method as described.^55, 56^ It was known that rats at this age had differentiated SCs similar to those of the adults and less germ cells.^55^ For different assays subsequently, the isolated SCs were plated at different densities on Matrigel-coated culture dishes: six-well plates at 0.5 × 10^6^/cm^2^ for immunoblot, immunoprecipitation, and endocytosis assays; coverslips at 0.05 × 10^6^/cm^2^ for immunofluorescence microscopy; Millicell-HA inserts (Millipore, Boston, MA, USA) at 1.2 × 10^6^/cm^2^ for TER measurement and TJ permeability barrier assessment. The primary SCs were cultured in serum-free DMEM/F12 (containing 2.5 ng/ml epidermal growth factor, 10 *μ*g/ml insulin, 5 *μ*g/ml transferrin, and 5 *μ*g/ml bacitracin) at 35 °C in an atmosphere of 5% CO_2_ for 7 days with medium changed once a day. On day 2, cultured cells were subjected to a hypotonic treatment using 10 mM Tris (pH=7.4) at room temperature for 2 min to eliminate remaining germ cells. Therefore, the purity of these SC cultures were more than 98% with negligible Leydig, germ, and myoid cells. To determine the cytotoxicity of Aroclor1254 on primary SCs, cell viability assay was performed on day 3 using an Enhanced Cell Counting Kit-8 (Beyotime, Guangzhou, China), in which SCs were exposed to 0–80 *μ*g/ml Aroclor1254 (diluted in dimethylsulfoxide, DMSO) and terminated at 6, 12, 24, or 48 h.

In order to investigate the effects of Aroclor1254 on BTB function *in vitro* and the role of p38 MAPK pathway during the process, cultured SCs were subjected to Aroclor1254 at doses without detectable cytotoxity (5 or 10 *μ*g/ml) or SB203580 (10 *μ*M, a specific inhibitor of the p38 MAPK pathway), or SB203580 (10 *μ*M) plus Aroclor1254 (10 *μ*g/ml) from culture day 3 for 48 h with the vehicle DMSO (0.1%, v/v) as the control. On day 5, treated SCs were replenished with fresh DMEM/F12 medium with supplements.

To determine the involvement of caveolin-dependent endocytosis in the effects of Aroclor1254 on the SC barrier *in vitro*, CO (2 U/ml CO, a specific caveolin pathway inhibitor) was added to the culture medium from day 4 before Aroclor1254 (10 *μ*g/ml) treatment from day 5.

In each experiment, triplicate SC cultures were used per time point and each experiment was repeated at least three times using different batches of SCs except pilot experiments to ensure optimal treatment conditions.

### Transient transfection of small interfering RNA in cultured SCs

In experiments to assess the participation of Itch-mediated occludin degradation during the disruption of Aroclor1254 on the SC permeability barrier, cultured SCs were transiently transfected with siRNA specifically targeting rat Itch (sense: 5′-UAAAACCACCAUUUGAAAGGG-3′, antisense: 5′-CUUUCAAAUGGUGGUUUUAAG-3′) or non-targeting siRNA control (sense: 5′-AGGAGAUGUGAUAUUGGCUTT-3′, antisense: 5′-AGCCAAUAUCACAUCUCCUTT-3′) on day 3. The final concentrations of siRNA were 150 nM for TJ permeability assays, 100 nM for immunoblot or immunoprecipitation analysis, and 80 nM for immunofluorescence, based on pilot experiments for different cell densities with optimal phenotypes and non-detectable cytotoxicity. To indicate the success transfection, 1 nM siGLO-Red was co-transfected in immunofluorescence assays. SiRNAs were first mixed with the transfection reagent in Opti-MEM medium before transfection according to the manufacturer’s instruction. After transfection for 24 h, SCs were rinsed and replenished with fresh culture medium before Aroclor1254 treatment from day 5.

### Assessment of TJ permeability barrier *in vitro*

Functional TJ barriers of each treatment and control groups were assessed by measuring resistance (R) across the SC monolayer cultured on matrigel-coated millicell inserts using a Millicell ERS-2 Epithelial Volt-Ohm Meter from day 1 to day 7, which represented the ability of the SCs barrier to resist current passage between the two electrodes placed, respectively, in the basal and the apical chamber of the bicameral units. In each experiment, resistance of a cell-free unit was recorded simultaneously at each designated time point as the blank control. The TER value (Ω cm^2^) was calculated by the following formula: (*R*_unknown_−*R*_blank_) × culture area.

The permeability of the SC barrier of each group in different experiment was also evaluated by using sodium fluorescein (Na-F, mo.wt. 376 Da) as the marker. Specifically, culture medium in the basal chambers of the bicameral culture unit was replaced with the permeability assay buffer (136 mM NaCl, 2.7 mM KCl, 0.9 mM CaCl_2_, 0.5 mM MgCl_2_, 1.5 mM KH_2_PO_4_, 10 mM NaH_2_PO_4_, 10 mM HEPES, and 25 mM glucose, pH 7.4), while the medium in the upper inserts was replaced with the same assay buffer containing 10 *μ*g/ml Na-F. After 1 h incubation at 35 °C, 50 *μ*l samples were retrieved from the basal chambers and subjected to analysis at 535 nm for Na-F emission. The calculated Na-F concentration in the basal chamber of the control group before treatment in each experiment was set as 100% arbitrarily.

### Immunoblot analysis

Lysates from testis or SCs were acquired by treating tissue homogenate or cells with freshly prepared IP lysis buffer (50 mM Tris, 150 mM NaCl, 2 mM EGTA, 10% glycerol, 1% Nonidet P-40, 2 mM phenylmethylsulphonyl fluoride, 1 mM sodium orthovanadate, 2 mM N-ethylmaleimide, 1 mg/ml leupeptin, and 1 mg/ml aprotinin, pH 7.4). After sonication and centrifugation at 15 000  × *g* for 1 h at 4 °C, clear supernatants were obtained and stored at −80 °C until use. Protein concentration of each sample was determined using the Bio-Rad DC protein assay kit (Bio-Rad Laboratories, Hercules, CA, USA). Approximately 20 *μ*g protein from SC or 40 *μ*g protein from testis were loaded per lane and resolved by SDS-PAGE for immunoblot using specific primary antibodies with reduced GAPDH serving as a loading control. Enhanced chemiluminescence was applied using a kit from Pierce Chemical Company according to the manufacturer’s instructions. For the *in vivo* study, testis tissue within 4 mm from the Aroclor1254 entry point was selected. To avoid inter-experimental variations, all samples from an independent experiment were analyzed simultaneously by immunoblot.

### Immunoprecipitation for ubiquitination assay

Co-IP was performed to detect ubiquitination level of TJ protein occludin in Aroclor1254-treated SCs with or without SB203580, or after Itch knockdown by siRNA in this study. During the 48 h treatment of Aroclor1254, MG-132 (50 *μ*M) was added for 60 min every 24 h to inhibit proteasome degradation of ubiquitine-conjugated proteins in SCs. To be specific, 500 *μ*g protein lysate from each sample was pre-incubated with 2 *μ*g normal rabbit IgG for 1 h to avoid non-specific interactions. Then the lysate was added with 15 *μ*l protein A/G plus agarose beads before incubation for another 1 h. After concentration at 1000 × *g* for 5 min, the supernatant was collected and subsequently incubated with 2.5 *μ*g rabbit anti-occludin antibody overnight at room temperature. After that, 30 *μ*l protein A/G plus agarose beads were applied to precipitate the immunocomplexes for 2 h, which were collected by centrifugation at 1000  × *g* for 5 min and washed three to four times with IP lysis buffer. After boiling and centrifugation, agarose beads were pelleted and the supernatants were resolved by SDS-PAGE and subjected to immunoblots using anti-ubiquitine antibody.

### Endocytosis assay

Endocytosis assays for junction proteins were performed according to the method published previously with minor modifications.^7, 57^ Briefly, cultured SCs on day 4 were treated with Sulfo-NHS-SS-Biotin (0.5 mg/ml) in biotinylation buffer (PBS containing 0.33 mM MgCl_2_ and 0.9 mM CaCl_2_) at 4 °C for 30 min for biotinylation of membrane proteins, followed by a 15 min quenching at 4 °C using 50 mM ammonium chloride in the biotinylation buffer to deactivate unbonded biotin. Then, the endocytosis was induced by incubating SCs at 35 °C with DMEM/F12 containing Aroclor1254 with or without SB203580 (or CO), which were terminated at different time points (15, 30, 60 min) by stripping the residual biotin (not internalized) on the cell membrane with 50 mM sodium 2-mercaptoethanesulfonate (MESNA, in 100 mM Tris/HCl containing 100 mM NaCl, 2.5 mM CaCl_2_) for 30 min at 4 °C, followed subsequently with a 15 min quenching by 5 mg/ml iodoacetamide in the biotinylation buffer. Thereafter, SC lysates were prepared with fresh IP lysis buffer as described above and 500 *μ*g total protein of each sample was subjected to UltraLink Immobilized NeutrAvidin Plus Resin for pulling down of biotinylated proteins overnight at 4 °C. Then immunoblots were performed using specific antibodies targeting at different endocytosed cell junction proteins. Total biotinylated protein at 0 min without stripping served as a positive control.

### Immunofluorescence microscopy

Bouin’s fixative or paraformaldehyde (4%, freshly diluted in PBS) was used to fix frozen testes sections (7 *μ*m) on poly-l-lysine-coated slides or SCs cultured on coverslips for 5 min, respectively. Then, fixed sections or SCs were permeabilized with Triton X-100 (1%, in PBS) for 4 min before being blocked with 5% BSA for 40 min. After aspirating the blocking liquid without washing, sections or cells were incubated with primary antibodies (1 : 200, diluted in 1% BSA) overnight at 4 °C or at room temperature, respectively. After washing, secondary antibody conjugated with Alexa Fluor 488 or Alexa Fluor 555 (1 : 150, diluted in 1% BSA) was added to the samples and incubated at room temperature for another 1 h. At last, the sections or coverslips were mounted with Prolong Gold Antifade reagent containing 4, 6-diamidino-2-phenylindole (DAPI) and observed under an Olympus BX60 fluorescence microscope (Olympus, Tokyo, Japan). Images from different groups were captured by a SpotRT digital camera in TIFF format using the same exposure time and adjusted for overlay by Photoshop (Adobe, San Jose, CA, USA). All the micrographs exhibited herein are representative pictures of at least three independent experiments.

### Quantitative real-time PCR analysis

Total RNA was isolated from SCs using Trizol reagent and then reverse transcribed into cDNAs using M-MLV reverse transcriptase. Real-time PCR was performed using the QuantiTect SYBR Green PCR Kit with primers specific to rat occludin (sense: 5′-taatgggagtcaacccgact-3′, antisense: 5′-tgaaccccaggacaatggct-3′), GAPDH co-amplification (sense: 5′-gctggtcatcaacgggaaac-3′, antisense: 5′-ggtgaagacgccagtagac-3′) serving as the internal control. The mRNA level of occludin was then calculated by the method of 2^−^^ΔΔCT^.

### Statistical analysis

All experiments were performed three to four times using different adult Sprague–Dawley rats or SCs isolated from different batches of 20-day-old rats. GB-STAT statistical software (version 7.0, Dynamic Microsystems, Inc., Silver Spring, MD, USA) was applied in analysis of data from the BTB integrity assay, immunoblot, immunoprecipitation, TER and TJ barrier permeability assays, and endocytosis assay in this study. Statistical significance was analyzed with one-way ANOVA or Student’s *t-*test before a two-tailed Dunnett’s test.
