# Supplementary material for: Aroclor1254 disrupts the blood–testis barrier by promoting endocytosis and degradation of junction proteins via p38 MAPK pathway
Source: Cell Death Dis. 2017 May 25;8(5):e2823–. doi: 10.1038/cddis.2017.224 (PMC5520738; doi:10.1038/cddis.2017.224)
Supplement: Supplementary Figure legends [file cddis2017224x1.pdf]

Supplementary Material

Supplementary Figure legends

Supplementary Figure 1 Changes of junction proteins distribution in rat testis after Aroclor1254 administration. Testis cryosections from different groups of rats were used for immunofluorescence analysis. The localization of BTB proteins were shown by Alexa Fluor 555-conjugated secondary antibody (*red*) with cell nuclei stained with DAPI (*blue*). An attenuated signal of occludin (denoted by the *white arrows*) and "broader" signals of JAM-A and N-cadherin (denoted by the *white brackets*) were observed in the Aroclor1254 group as compared with the vehicle control, which were not shown in the Aroclor1254+SB203580 group. Scale bar=40 μm, which applied to all micrographs in Figure S1.

Supplementary Figure 2 Aroclor1254 did not alter Ser473 and Thr308 phosphorylation of PKB in cultured SCs. (**a**) Immunoblot analysis to assess the levels of PKB phosphorylation in SC lysates prepared at different time points after Aroclor1254 treatment. (**b**) Histograms summarizing the result shown in (a) after the level of phosphorylated PKB (p-PKB) was normalized to total PKB expression. Protein level of the vehicle control group at time 0 was arbitrarily set at 1. Each bar refers to mean±SD of n=3 experiments using different batches of SC lysates.

Supplementary Figure 3 Aroclor1254 did not alter the endocytosis and mRNA level of occludin in cultured SCs. (**a**) Immunoblot analysis of endocytosed occludin in SCs at different time points after cell surface biotinylation in the presence of Aroclor1254 (or the mixture of Aroclor1254 and SB203580). The endocytosed proteins were pulled-down by UltraLink

Immobilized NeutrAvidin Plus Resin. Total biotinylated proteins at 0 min without stripping were detected as a positive control. Cell lysates without pull-down were also analyzed by immunoblot to show the steady-state level of occludin with GAPDH served as the loading control. (**b**) Line and scatter graphs summarizing the result shown in (a) by calculating the percentage of endocytosed protein versus the total biotinylated protein, which was then normalized with the steady-state level of occludin at each time point. (**c**) qPCR analysis of occludin mRNA level in SCs after Aroclor1254 treatment for 48 h. Each bar refers to mean±SD of n=3 independent experiments using SCs cultured from different rats.

Supplementary Figure 4 The effects of Aroclor1254 on BTB protein levels in the presence of cholesterol oxidase. (**a**) A schematic drawing illustrating the treatment regimen for both FigureS2 and Figure5. CO was added to the SC culture medium at 2 U/ml from day 4. 24 hours later, randomly selected cultures were treated with 10 μg/ml Aroclor1254 for another 48 h. (**b**) Immunoblot analysis to assess the BTB protein levels in SC lysates on day 7 after treatment of Aroclor1254 or CO+Aroclor1254, with GAPDH serving as a protein loading control. (**c**) Histograms illustrating the result shown in (b) after each data point was normalized against GAPDH except that the level of phosphorylated p38 MAPK (p-p38) was normalized to total p38 MAPK expression. The protein level in the vehicle control group was arbitrarily set at 1. **, *P*<0.01, compared with the vehicle control group. $^{\lozenge\lozenge}$, *P*<0.01, compared with the CO-treated cells.

Supplementary Figure 5 The effects of Aroclor1254 on BTB protein levels after Itch-silencing by siRNA. (**a**) A schematic drawing illustrating the treatment regimen for both FigureS3 and Figure6.

The siRNA targeting at rat Itch or the non-targeting control siRNA was transfected into SCs on culture day 3 for 24 hours. After another 24-hour interval, the SCs were treated with Aroclor1254 from day 5 for 48 h. (**b**) Immunoblot analysis to detect the BTB protein levels in SC lysates harvested on day 7 after treatment, with GAPDH serving as a protein loading control. The knock-down efficiency of Itch siRNA was found to be approximately 80%. (**c**) Histograms illustrating the result shown in (b) after each data point was normalized against GAPDH except that the level of phosphorylated p38 MAPK (p-p38) was normalized to total p38 MAPK expression. The protein level in the control siRNA transfected SCs was arbitrarily set at 1. No significant difference in occludin level was detected after Aroclor1254 treatment in Itch-silencing cells. **, *P*<0.01, compared with the control siRNA group. $^{\lozenge\lozenge}$, *P*<0.01, compared with the Itch siRNA group. *N.S.*, no significant difference between two indicated groups.
