# Supplementary figures and images for: Aroclor1254 disrupts the blood–testis barrier by promoting endocytosis and degradation of junction proteins via p38 MAPK pathway
Source: Cell Death Dis. 2017 May 25;8(5):e2823–. doi: 10.1038/cddis.2017.224 (PMC5520738; doi:10.1038/cddis.2017.224)

Fig.S1 (Jia XY, et al)

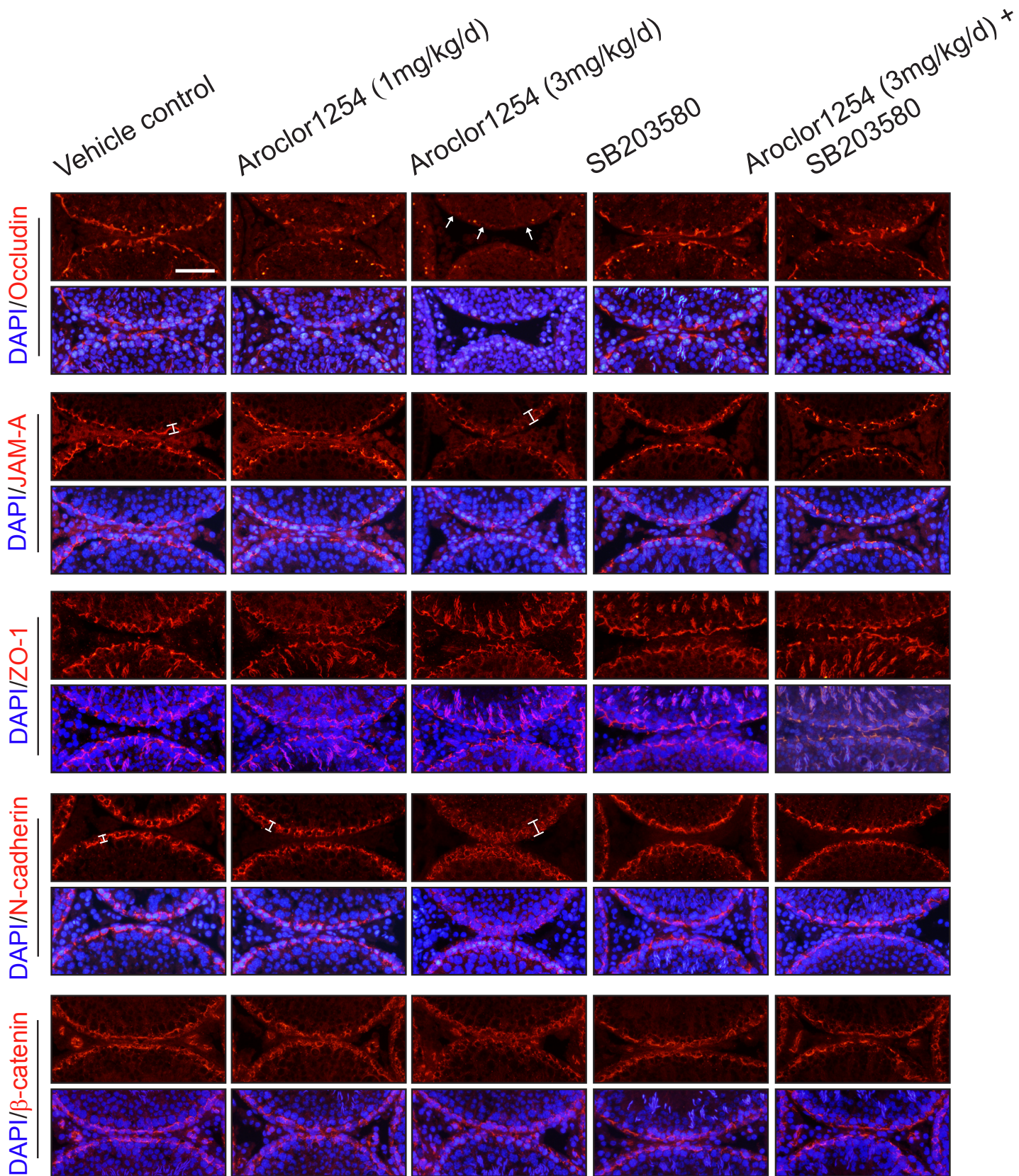

Supplement: Supplementary Figure 1 [file cddis2017224x2.pdf]

Fig.S2 (Jia XY, et al)

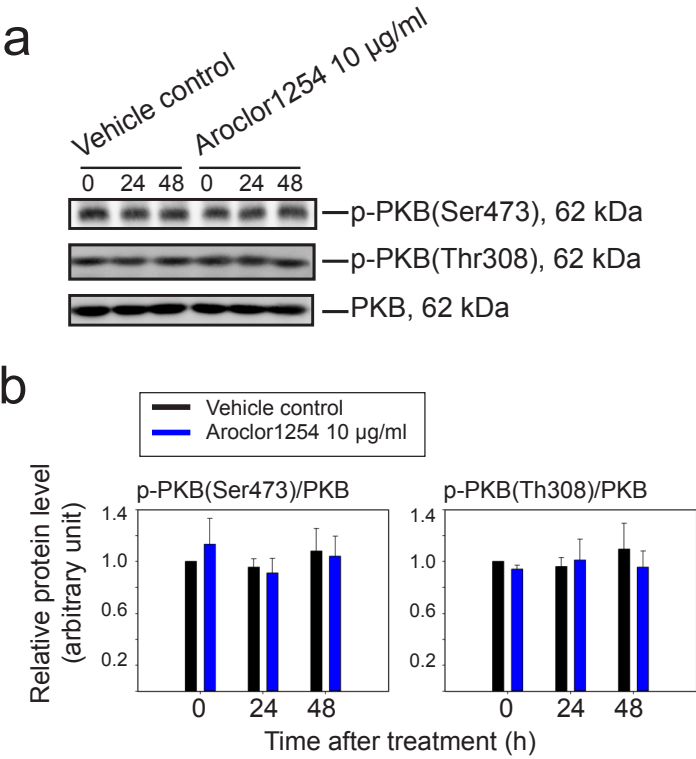

Supplement: Supplementary Figure 2 [file cddis2017224x3.pdf]

Fig.S3 (*Jia XY, et al*)

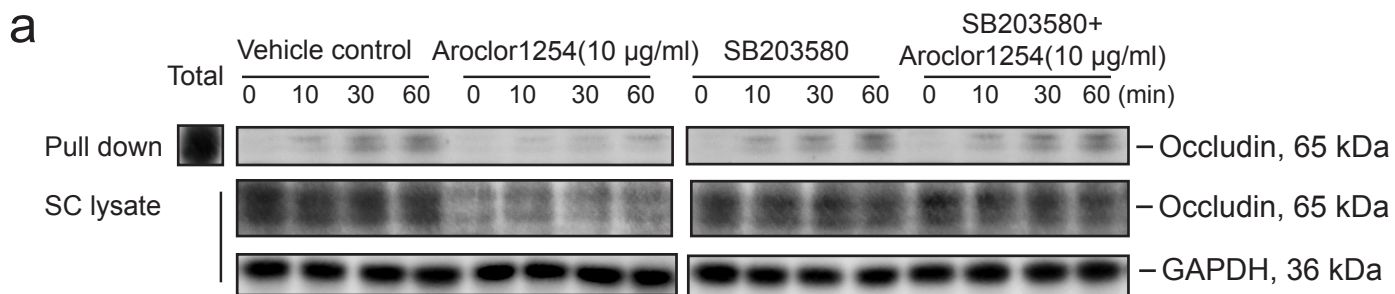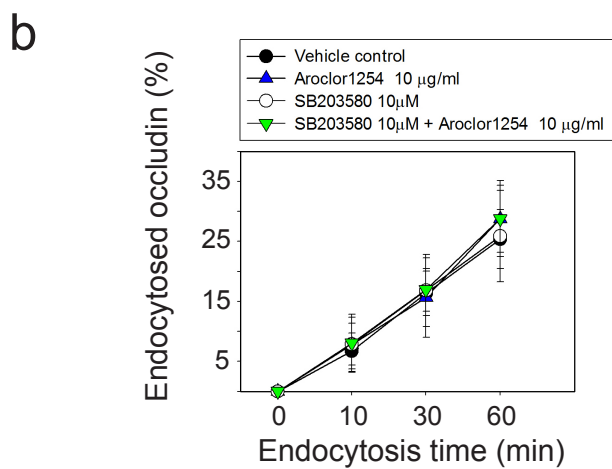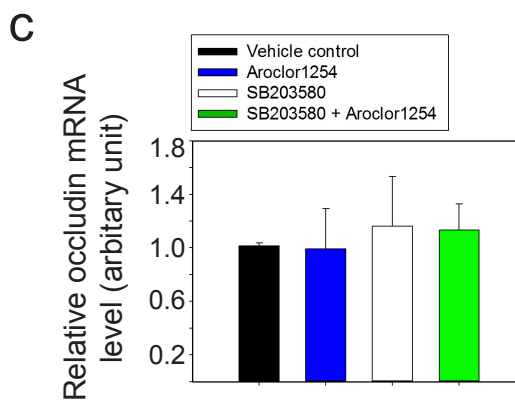

Supplement: Supplementary Figure 3 [file cddis2017224x4.pdf]

Fig.S4 (*Jia XY, et al*)

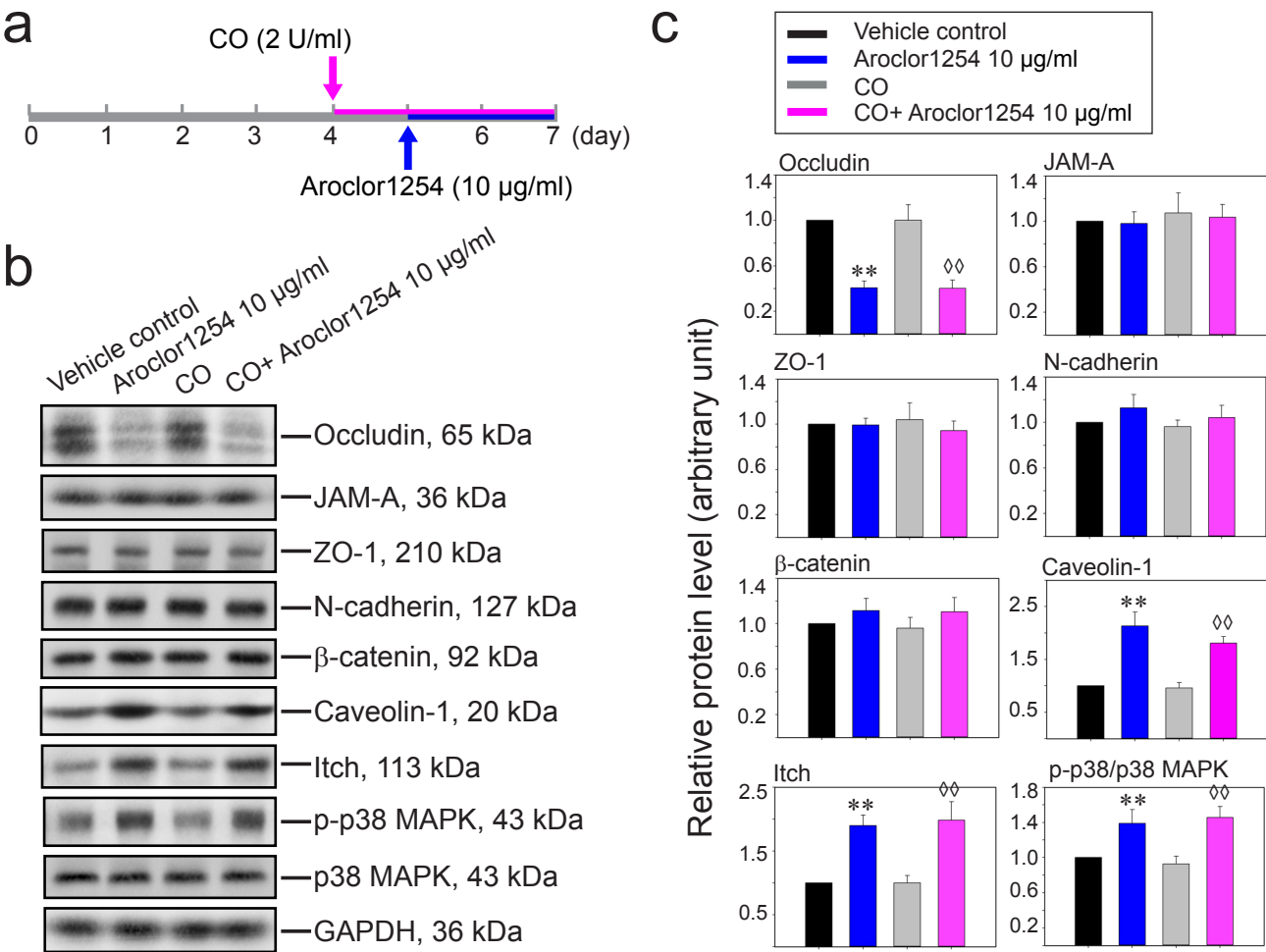

Supplement: Supplementary Figure 4 [file cddis2017224x5.pdf]

Fig.S5 (Jia XY, et al)

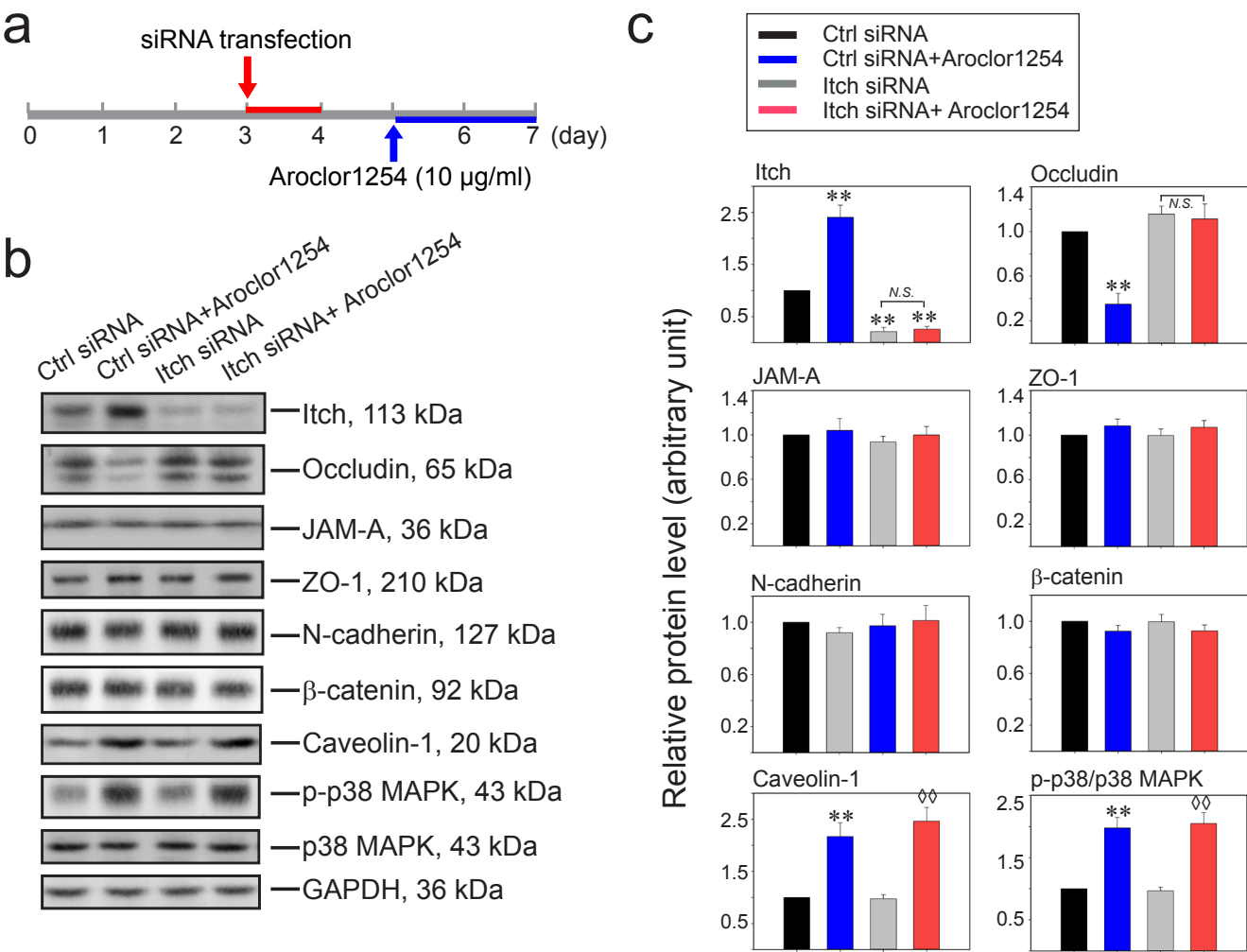

Supplement: Supplementary Figure 5 [file cddis2017224x6.pdf]
